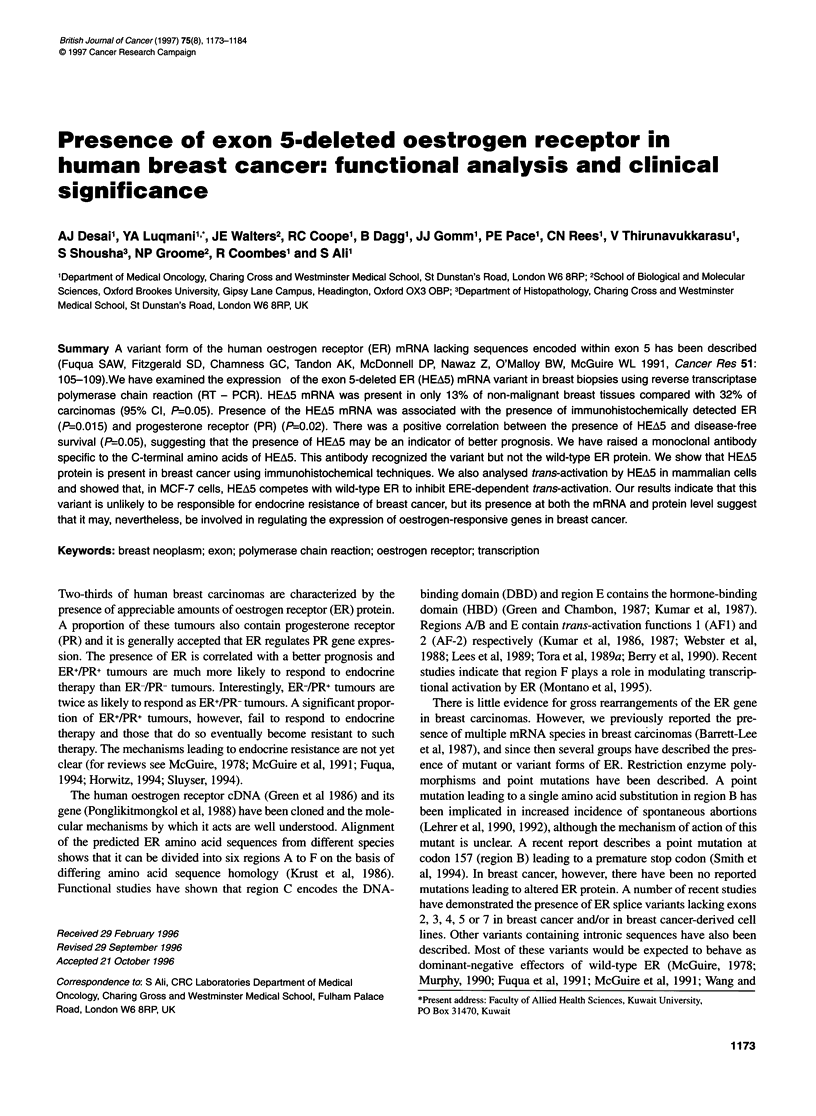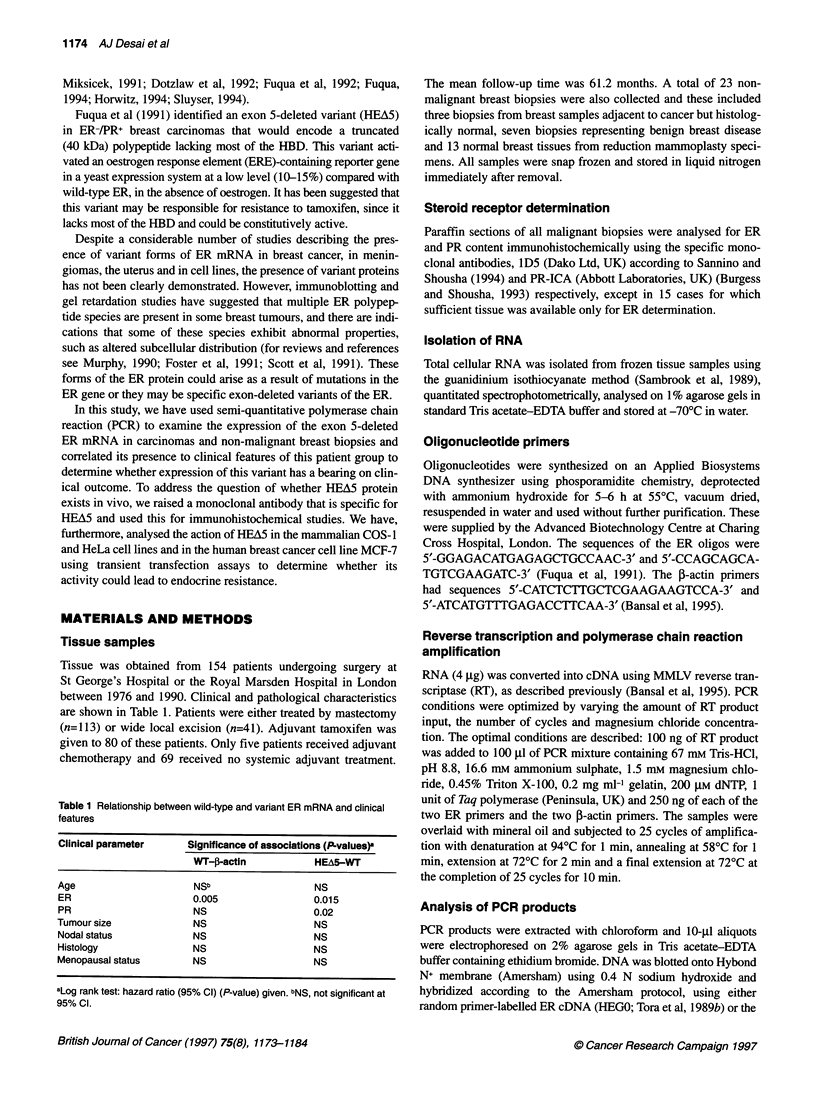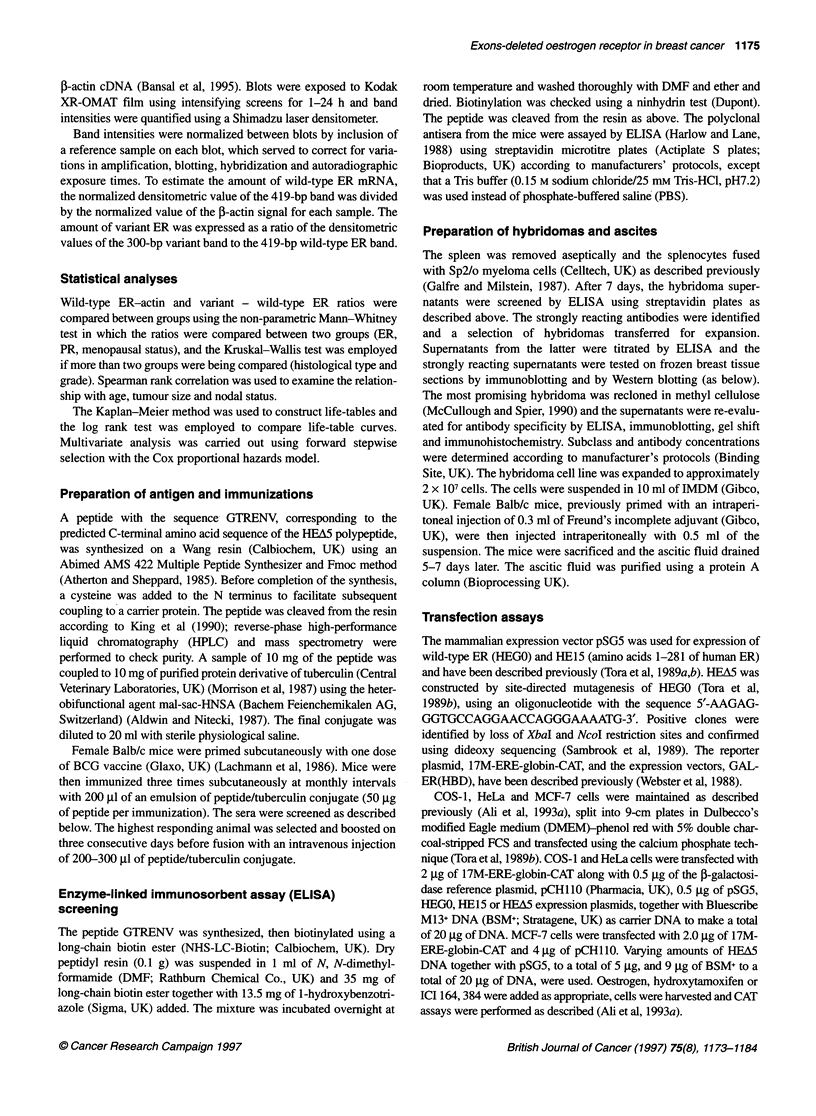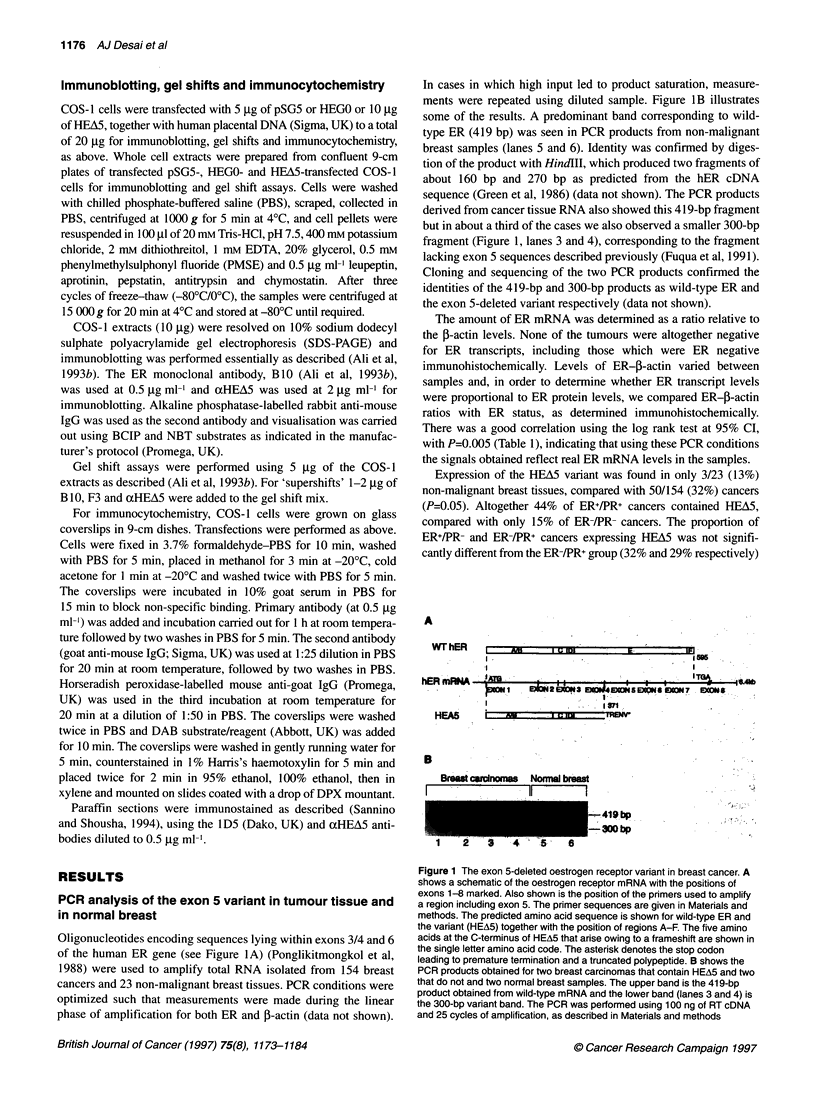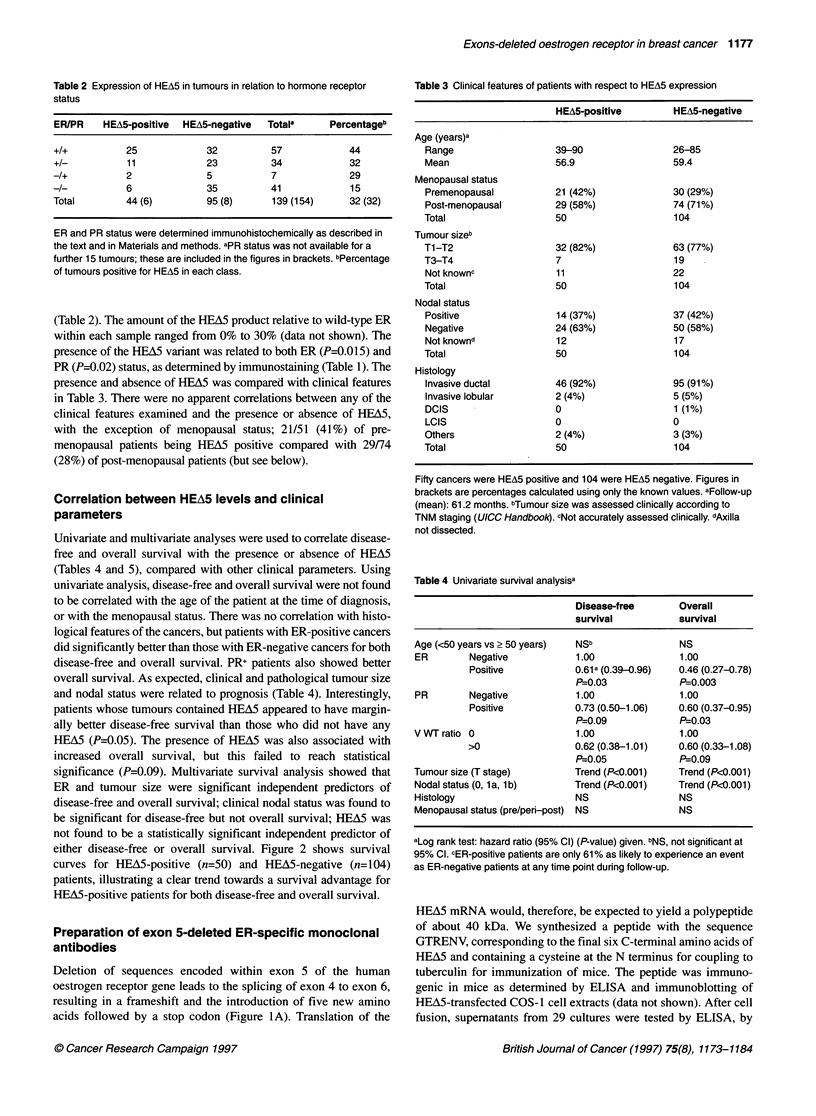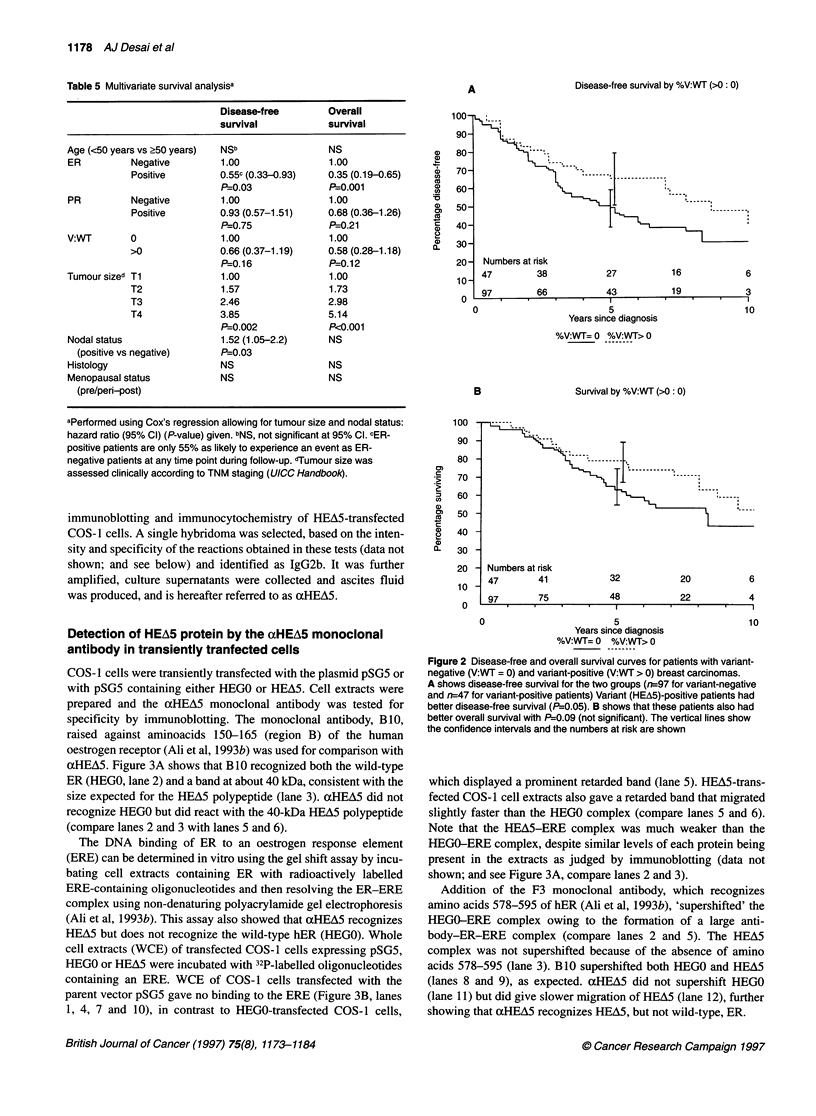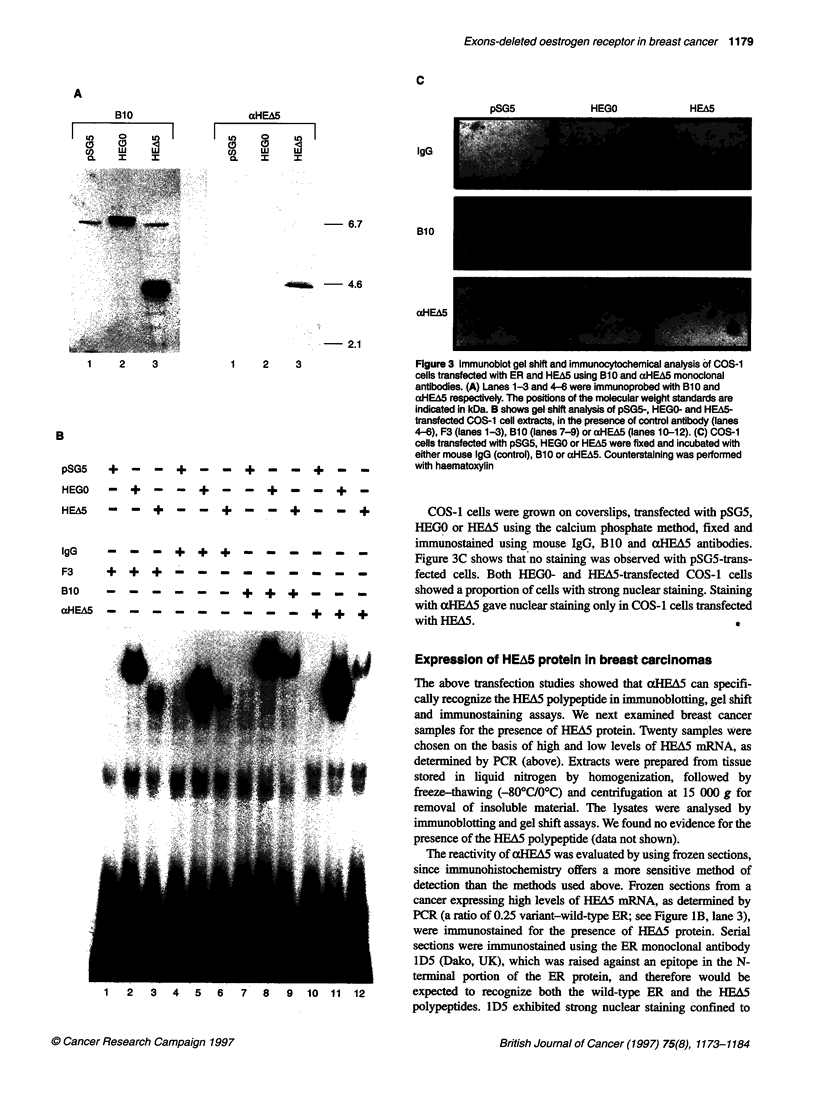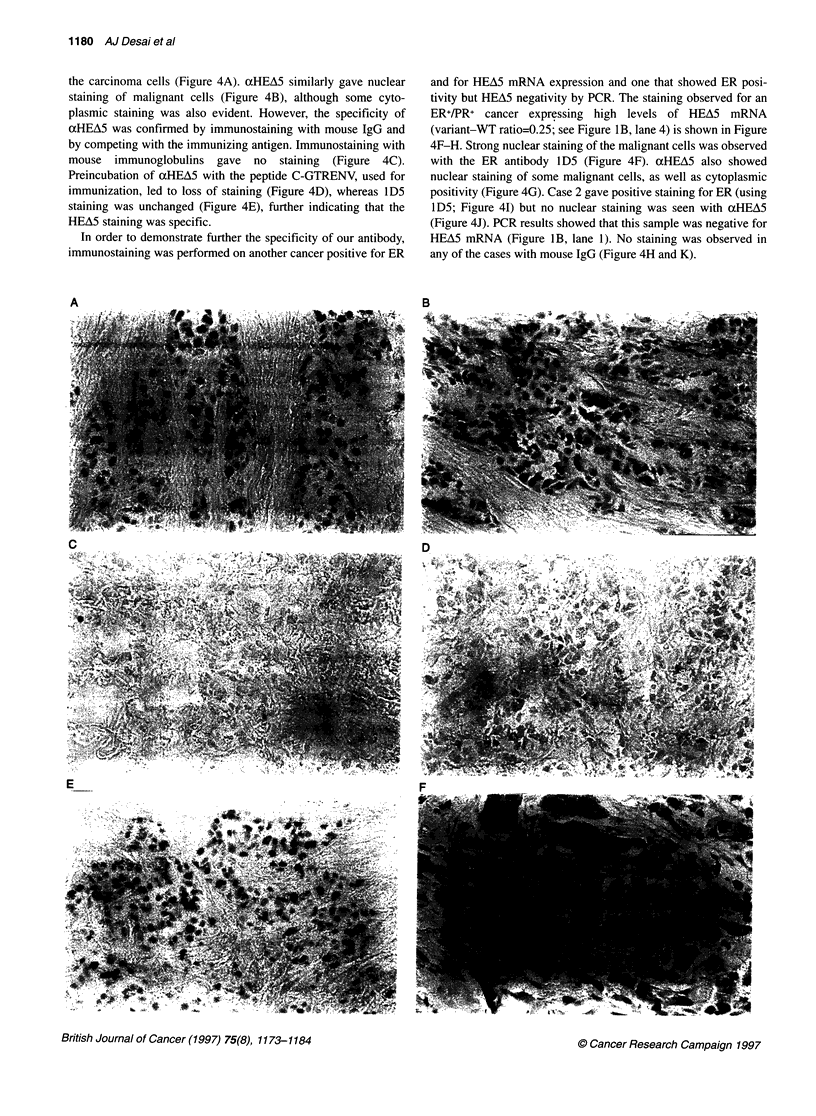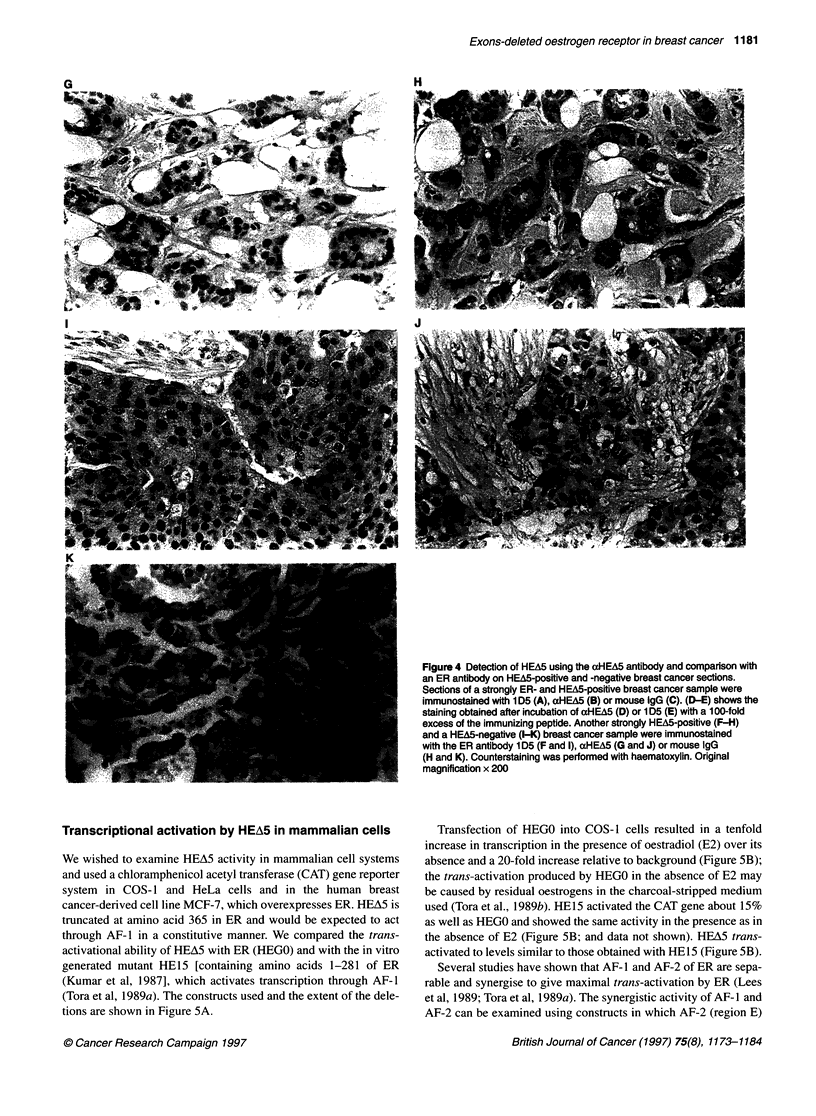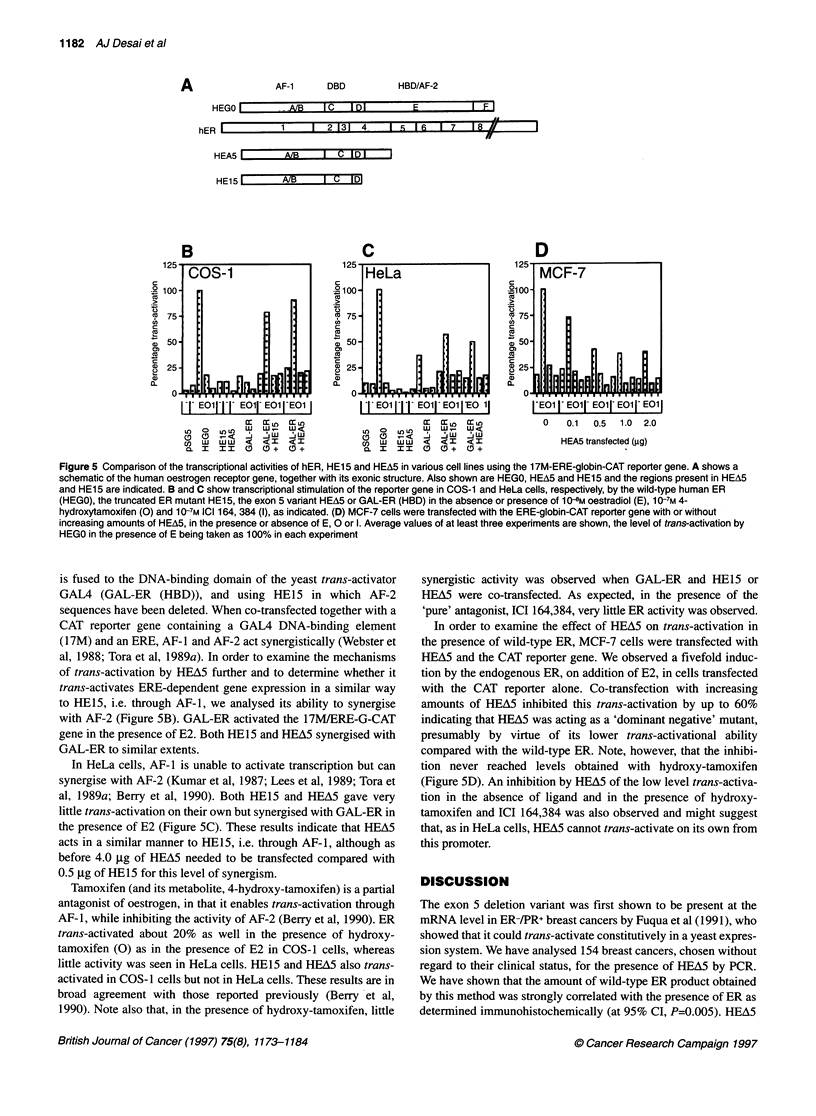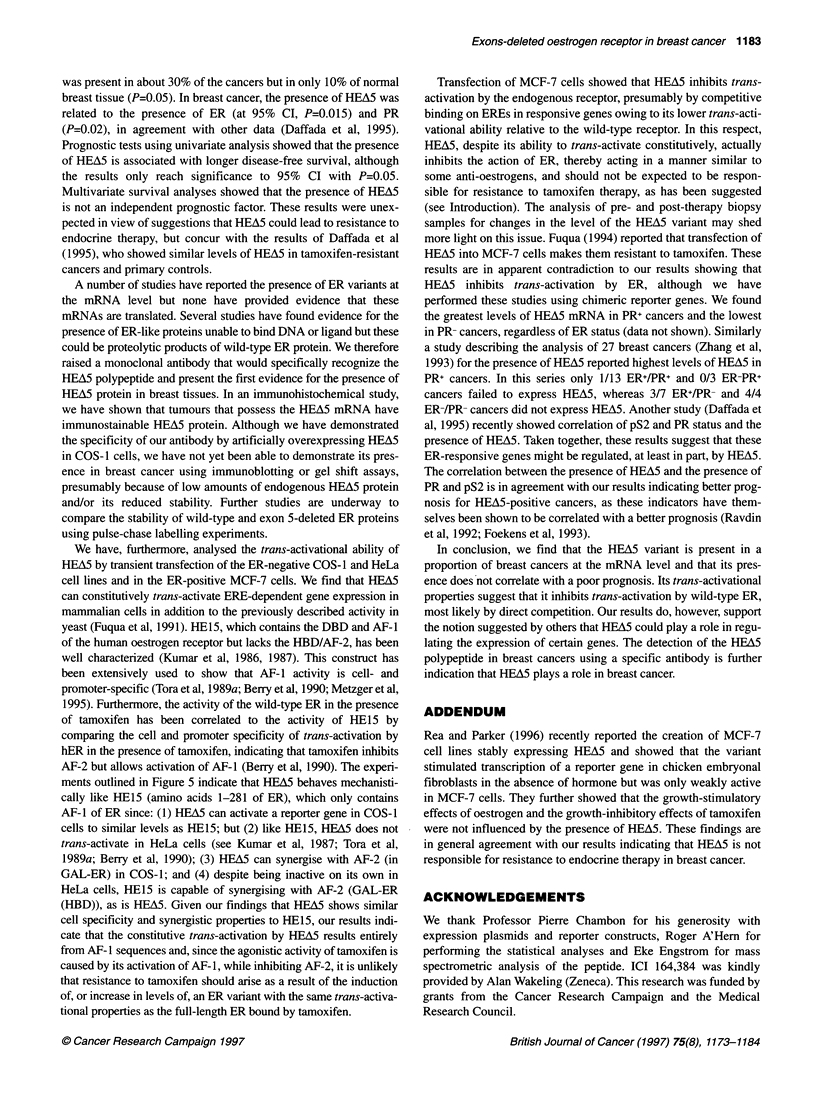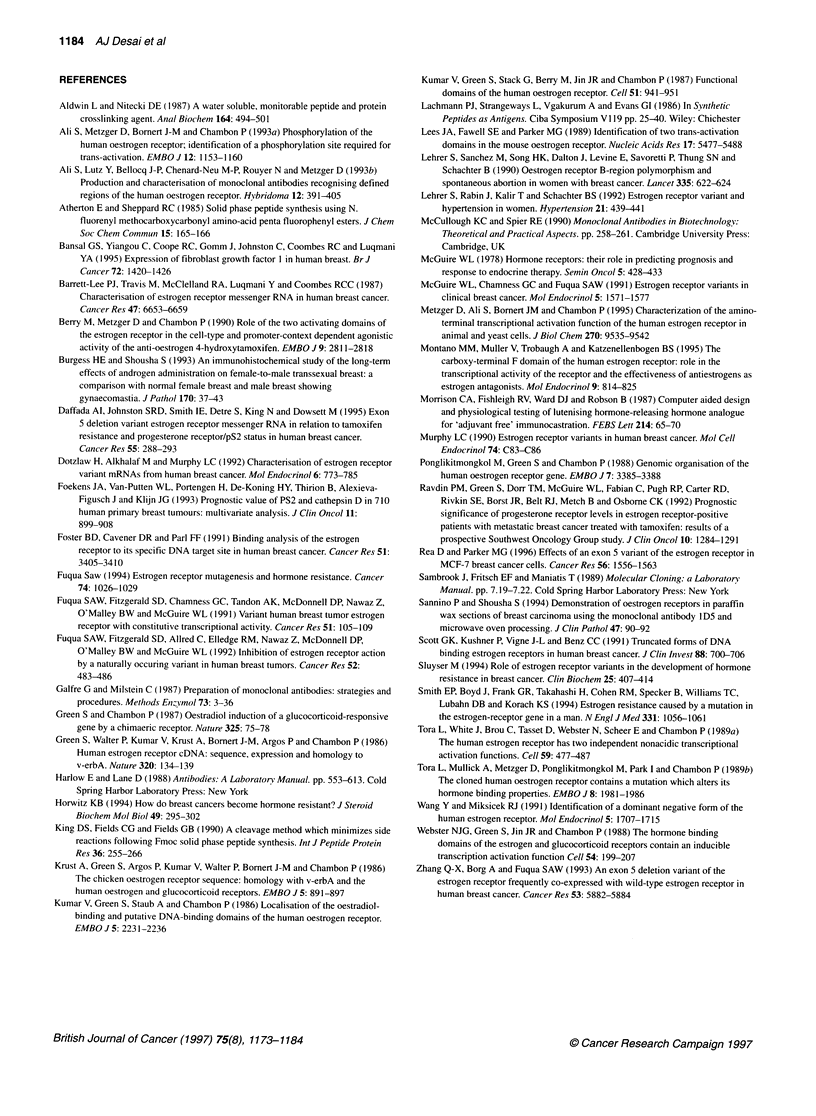# Presence of exon 5-deleted oestrogen receptor in human breast cancer: functional analysis and clinical significance.

**DOI:** 10.1038/bjc.1997.202

**Published:** 1997

**Authors:** A. J. Desai, Y. A. Luqmani, J. E. Walters, R. C. Coope, B. Dagg, J. J. Gomm, P. E. Pace, C. N. Rees, V. Thirunavukkarasu, S. Shousha, N. P. Groome, R. Coombes, S. Ali

**Affiliations:** Department of Medical Oncology, Charing Cross and Westminster Medical School, London, UK.

## Abstract

**Images:**


British Journal of Cancer (1997) 75(8), 1173–1184
© 1997 Cancer Research Campaign

### Presence of exon 5-deleted oestrogen receptor in human breast cancer: functional analysis and clinical significance

**AJ Desai[1], YA Luqmani[1,*], JE Walters[2], RC Coope[1], B Dagg[1], JJ Gomm[1], PE Pace[1], CN Rees[1], V Thirunavukkarasu[1], S Shousha[3], NP Groome[2], R Coombes[1] and S Ali[1]**

[1]Department of Medical Oncology, Charing Cross and Westminster Medical School, St Dunstan's Road, London W6 8RP; [2]School of Biological and Molecular Sciences, Oxford Brookes University, Gipsy Lane Campus, Headington, Oxford OX3 OBP; [3]Department of Histopathology, Charing Cross and Westminster Medical School, St Dunstan's Road, London W6 8RP, UK

**Summary** A variant form of the human oestrogen receptor (ER) mRNA lacking sequences encoded within exon 5 has been described (Fuqua SAW, Fitzgerald SD, Chamness GC, Tandon AK, McDonnell DP, Nawaz Z, O'Malloy BW, McGuire WL 1991, *Cancer Res* **51**: 105–109).We have examined the expression of the exon 5-deleted ER (HEΔ5) mRNA variant in breast biopsies using reverse transcriptase polymerase chain reaction (RT – PCR). HEΔ5 mRNA was present in only 13% of non-malignant breast tissues compared with 32% of carcinomas (95% CI, $P$=0.05). Presence of the HEΔ5 mRNA was associated with the presence of immunohistochemically detected ER ($P$=0.015) and progesterone receptor (PR) ($P$=0.02). There was a positive correlation between the presence of HEΔ5 and disease-free survival ($P$=0.05), suggesting that the presence of HEΔ5 may be an indicator of better prognosis. We have raised a monoclonal antibody specific to the C-terminal amino acids of HEΔ5. This antibody recognized the variant but not the wild-type ER protein. We show that HEΔ5 protein is present in breast cancer using immunohistochemical techniques. We also analysed *trans*-activation by HEΔ5 in mammalian cells and showed that, in MCF-7 cells, HEΔ5 competes with wild-type ER to inhibit ERE-dependent *trans*-activation. Our results indicate that this variant is unlikely to be responsible for endocrine resistance of breast cancer, but its presence at both the mRNA and protein level suggest that it may, nevertheless, be involved in regulating the expression of oestrogen-responsive genes in breast cancer.

**Keywords:** breast neoplasm; exon; polymerase chain reaction; oestrogen receptor; transcription

Two-thirds of human breast carcinomas are characterized by the presence of appreciable amounts of oestrogen receptor (ER) protein. A proportion of these tumours also contain progesterone receptor (PR) and it is generally accepted that ER regulates PR gene expression. The presence of ER is correlated with a better prognosis and $ER^+/PR^+$ tumours are much more likely to respond to endocrine therapy than $ER^-/PR^-$ tumours. Interestingly, $ER^-/PR^+$ tumours are twice as likely to respond as $ER^+/PR^-$ tumours. A significant proportion of $ER^+/PR^+$ tumours, however, fail to respond to endocrine therapy and those that do so eventually become resistant to such therapy. The mechanisms leading to endocrine resistance are not yet clear (for reviews see McGuire, 1978; McGuire et al, 1991; Fuqua, 1994; Horwitz, 1994; Sluyser, 1994).

The human oestrogen receptor cDNA (Green et al 1986) and its gene (Ponglikitmongkol et al, 1988) have been cloned and the molecular mechanisms by which it acts are well understood. Alignment of the predicted ER amino acid sequences from different species shows that it can be divided into six regions A to F on the basis of differing amino acid sequence homology (Krust et al, 1986). Functional studies have shown that region C encodes the DNA-binding domain (DBD) and region E contains the hormone-binding domain (HBD) (Green and Chambon, 1987; Kumar et al, 1987). Regions A/B and E contain *trans*-activation functions 1 (AF1) and 2 (AF-2) respectively (Kumar et al, 1986, 1987; Webster et al, 1988; Lees et al, 1989; Tora et al, 1989*a*; Berry et al, 1990). Recent studies indicate that region F plays a role in modulating transcriptional activation by ER (Montano et al, 1995).

There is little evidence for gross rearrangements of the ER gene in breast carcinomas. However, we previously reported the presence of multiple mRNA species in breast carcinomas (Barrett-Lee et al, 1987), and since then several groups have described the presence of mutant or variant forms of ER. Restriction enzyme polymorphisms and point mutations have been described. A point mutation leading to a single amino acid substitution in region B has been implicated in increased incidence of spontaneous abortions (Lehrer et al, 1990, 1992), although the mechanism of action of this mutant is unclear. A recent report describes a point mutation at codon 157 (region B) leading to a premature stop codon (Smith et al, 1994). In breast cancer, however, there have been no reported mutations leading to altered ER protein. A number of recent studies have demonstrated the presence of ER splice variants lacking exons 2, 3, 4, 5 or 7 in breast cancer and/or in breast cancer-derived cell lines. Other variants containing intronic sequences have also been described. Most of these variants would be expected to behave as dominant-negative effectors of wild-type ER (McGuire, 1978; Murphy, 1990; Fuqua et al, 1991; McGuire et al, 1991; Wang and

*Received 29 February 1996*
*Revised 29 September 1996*
*Accepted 21 October 1996*

*Correspondence to*: S Ali, CRC Laboratories Department of Medical Oncology, Charing Gross and Westminster Medical School, Fulham Palace Road, London W6 8RP, UK

*Present address: Faculty of Allied Health Sciences, Kuwait University, PO Box 31470, Kuwait

Miksicek, 1991; Dotzlaw et al, 1992; Fuqua et al, 1992; Fuqua, 1994; Horwitz, 1994; Sluyser, 1994).

Fuqua et al (1991) identified an exon 5-deleted variant (HEΔ5) in $ER^-/PR^+$ breast carcinomas that would encode a truncated (40 kDa) polypeptide lacking most of the HBD. This variant activated an oestrogen response element (ERE)-containing reporter gene in a yeast expression system at a low level (10–15%) compared with wild-type ER, in the absence of oestrogen. It has been suggested that this variant may be responsible for resistance to tamoxifen, since it lacks most of the HBD and could be constitutively active.

Despite a considerable number of studies describing the presence of variant forms of ER mRNA in breast cancer, in meningiomas, the uterus and in cell lines, the presence of variant proteins has not been clearly demonstrated. However, immunoblotting and gel retardation studies have suggested that multiple ER polypeptide species are present in some breast tumours, and there are indications that some of these species exhibit abnormal properties, such as altered subcellular distribution (for reviews and references see Murphy, 1990; Foster et al, 1991; Scott et al, 1991). These forms of the ER protein could arise as a result of mutations in the ER gene or they may be specific exon-deleted variants of the ER.

In this study, we have used semi-quantitative polymerase chain reaction (PCR) to examine the expression of the exon 5-deleted ER mRNA in carcinomas and non-malignant breast biopsies and correlated its presence to clinical features of this patient group to determine whether expression of this variant has a bearing on clinical outcome. To address the question of whether HEΔ5 protein exists in vivo, we raised a monoclonal antibody that is specific for HEΔ5 and used this for immunohistochemical studies. We have, furthermore, analysed the action of HEΔ5 in the mammalian COS-1 and HeLa cell lines and in the human breast cancer cell line MCF-7 using transient transfection assays to determine whether its activity could lead to endocrine resistance.

### MATERIALS AND METHODS

### Tissue samples

Tissue was obtained from 154 patients undergoing surgery at St George's Hospital or the Royal Marsden Hospital in London between 1976 and 1990. Clinical and pathological characteristics are shown in Table 1. Patients were either treated by mastectomy (*n*=113) or wide local excision (*n*=41). Adjuvant tamoxifen was given to 80 of these patients. Only five patients received adjuvant chemotherapy and 69 received no systemic adjuvant treatment. The mean follow-up time was 61.2 months. A total of 23 non-malignant breast biopsies were also collected and these included three biopsies from breast samples adjacent to cancer but histologically normal, seven biopsies representing benign breast disease and 13 normal breast tissues from reduction mammoplasty specimens. All samples were snap frozen and stored in liquid nitrogen immediately after removal.

**Table 1** Relationship between wild-type and variant ER mRNA and clinical features

| Clinical parameter | Significance of associations (*P*-values)[a] | |
|---|---|---|
| | WT-β-actin | HEΔ5-WT |
| Age | NS[b] | NS |
| ER | 0.005 | 0.015 |
| PR | NS | 0.02 |
| Tumour size | NS | NS |
| Nodal status | NS | NS |
| Histology | NS | NS |
| Menopausal status | NS | NS |

[a]Log rank test: hazard ratio (95% CI) (*P*-value) given. [b]NS, not significant at 95% CI.

### Steroid receptor determination

Paraffin sections of all malignant biopsies were analysed for ER and PR content immunohistochemically using the specific monoclonal antibodies, 1D5 (Dako Ltd, UK) according to Sannino and Shousha (1994) and PR-ICA (Abbott Laboratories, UK) (Burgess and Shousha, 1993) respectively, except in 15 cases for which sufficient tissue was available only for ER determination.

### Isolation of RNA

Total cellular RNA was isolated from frozen tissue samples using the guanidinium isothiocyanate method (Sambrook et al, 1989), quantitated spectrophotometrically, analysed on 1% agarose gels in standard Tris acetate–EDTA buffer and stored at –70°C in water.

### Oligonucleotide primers

Oligonucleotides were synthesized on an Applied Biosystems DNA synthesizer using phosporamidite chemistry, deprotected with ammonium hydroxide for 5–6 h at 55°C, vacuum dried, resuspended in water and used without further purification. These were supplied by the Advanced Biotechnology Centre at Charing Cross Hospital, London. The sequences of the ER oligos were 5′-GGAGACATGAGAGCTGCCAAC-3′ and 5′-CCAGCAGCATGTCGAAGATC-3′ (Fuqua et al, 1991). The β-actin primers had sequences 5′-CATCTCTTGCTCGAAGAAGTCCA-3′ and 5′-ATCATGTTTGAGACCTTCAA-3′ (Bansal et al, 1995).

### Reverse transcription and polymerase chain reaction amplification

RNA (4 μg) was converted into cDNA using MMLV reverse transcriptase (RT), as described previously (Bansal et al, 1995). PCR conditions were optimized by varying the amount of RT product input, the number of cycles and magnesium chloride concentration. The optimal conditions are described: 100 ng of RT product was added to 100 μl of PCR mixture containing 67 mM Tris-HCl, pH 8.8, 16.6 mM ammonium sulphate, 1.5 mM magnesium chloride, 0.45% Triton X-100, 0.2 mg $ml^{-1}$ gelatin, 200 μM dNTP, 1 unit of *Taq* polymerase (Peninsula, UK) and 250 ng of each of the two ER primers and the two β-actin primers. The samples were overlaid with mineral oil and subjected to 25 cycles of amplification with denaturation at 94°C for 1 min, annealing at 58°C for 1 min, extension at 72°C for 2 min and a final extension at 72°C at the completion of 25 cycles for 10 min.

### Analysis of PCR products

PCR products were extracted with chloroform and 10-μl aliquots were electrophoresed on 2% agarose gels in Tris acetate–EDTA buffer containing ethidium bromide. DNA was blotted onto Hybond $N^+$ membrane (Amersham) using 0.4 N sodium hydroxide and hybridized according to the Amersham protocol, using either random primer-labelled ER cDNA (HEG0; Tora et al, 1989*b*) or the

© Cancer Research Campaign 1997

β-actin cDNA (Bansal et al, 1995). Blots were exposed to Kodak XR-OMAT film using intensifying screens for 1–24 h and band intensities were quantified using a Shimadzu laser densitometer.

Band intensities were normalized between blots by inclusion of a reference sample on each blot, which served to correct for variations in amplification, blotting, hybridization and autoradiographic exposure times. To estimate the amount of wild-type ER mRNA, the normalized densitometric value of the 419-bp band was divided by the normalized value of the β-actin signal for each sample. The amount of variant ER was expressed as a ratio of the densitometric values of the 300-bp variant band to the 419-bp wild-type ER band.

### Statistical analyses

Wild-type ER–actin and variant – wild-type ER ratios were compared between groups using the non-parametric Mann–Whitney test in which the ratios were compared between two groups (ER, PR, menopausal status), and the Kruskal–Wallis test was employed if more than two groups were being compared (histological type and grade). Spearman rank correlation was used to examine the relationship with age, tumour size and nodal status.

The Kaplan–Meier method was used to construct life-tables and the log rank test was employed to compare life-table curves. Multivariate analysis was carried out using forward stepwise selection with the Cox proportional hazards model.

### Preparation of antigen and immunizations

A peptide with the sequence GTRENV, corresponding to the predicted C-terminal amino acid sequence of the HEΔ5 polypeptide, was synthesized on a Wang resin (Calbiochem, UK) using an Abimed AMS 422 Multiple Peptide Synthesizer and Fmoc method (Atherton and Sheppard, 1985). Before completion of the synthesis, a cysteine was added to the N terminus to facilitate subsequent coupling to a carrier protein. The peptide was cleaved from the resin according to King et al (1990); reverse-phase high-performance liquid chromatography (HPLC) and mass spectrometry were performed to check purity. A sample of 10 mg of the peptide was coupled to 10 mg of purified protein derivative of tuberculin (Central Veterinary Laboratories, UK) (Morrison et al, 1987) using the heterobifunctional agent mal-sac-HNSA (Bachem Feienchemikalen AG, Switzerland) (Aldwin and Nitecki, 1987). The final conjugate was diluted to 20 ml with sterile physiological saline.

Female Balb/c mice were primed subcutaneously with one dose of BCG vaccine (Glaxo, UK) (Lachmann et al, 1986). Mice were then immunized three times subcutaneously at monthly intervals with 200 μl of an emulsion of peptide/tuberculin conjugate (50 μg of peptide per immunization). The sera were screened as described below. The highest responding animal was selected and boosted on three consecutive days before fusion with an intravenous injection of 200–300 μl of peptide/tuberculin conjugate.

### Enzyme-linked immunosorbent assay (ELISA) screening

The peptide GTRENV was synthesized, then biotinylated using a long-chain biotin ester (NHS-LC-Biotin; Calbiochem, UK). Dry peptidyl resin (0.1 g) was suspended in 1 ml of *N*, *N*-dimethylformamide (DMF; Rathburn Chemical Co., UK) and 35 mg of long-chain biotin ester together with 13.5 mg of 1-hydroxybenzotriazole (Sigma, UK) added. The mixture was incubated overnight at room temperature and washed thoroughly with DMF and ether and dried. Biotinylation was checked using a ninhydrin test (Dupont). The peptide was cleaved from the resin as above. The polyclonal antisera from the mice were assayed by ELISA (Harlow and Lane, 1988) using streptavidin microtitre plates (Actiplate S plates; Bioproducts, UK) according to manufacturers' protocols, except that a Tris buffer (0.15 M sodium chloride/25 mM Tris-HCl, pH7.2) was used instead of phosphate-buffered saline (PBS).

### Preparation of hybridomas and ascites

The spleen was removed aseptically and the splenocytes fused with Sp2/o myeloma cells (Celltech, UK) as described previously (Galfre and Milstein, 1987). After 7 days, the hybridoma supernatants were screened by ELISA using streptavidin plates as described above. The strongly reacting antibodies were identified and a selection of hybridomas transferred for expansion. Supernatants from the latter were titrated by ELISA and the strongly reacting supernatants were tested on frozen breast tissue sections by immunoblotting and by Western blotting (as below). The most promising hybridoma was recloned in methyl cellulose (McCullough and Spier, 1990) and the supernatants were re-evaluated for antibody specificity by ELISA, immunoblotting, gel shift and immunohistochemistry. Subclass and antibody concentrations were determined according to manufacturer's protocols (Binding Site, UK). The hybridoma cell line was expanded to approximately $2 \times 10^7$ cells. The cells were suspended in 10 ml of IMDM (Gibco, UK). Female Balb/c mice, previously primed with an intraperitoneal injection of 0.3 ml of Freund's incomplete adjuvant (Gibco, UK), were then injected intraperitoneally with 0.5 ml of the suspension. The mice were sacrificed and the ascitic fluid drained 5–7 days later. The ascitic fluid was purified using a protein A column (Bioprocessing UK).

### Transfection assays

The mammalian expression vector pSG5 was used for expression of wild-type ER (HEG0) and HE15 (amino acids 1–281 of human ER) and have been described previously (Tora et al, 1989*a*,*b*). HEΔ5 was constructed by site-directed mutagenesis of HEG0 (Tora et al, 1989*b*), using an oligonucleotide with the sequence 5′-AAGAGGGTGCCAGGAACCAGGGAAAATG-3′. Positive clones were identified by loss of *Xba*I and *Nco*I restriction sites and confirmed using dideoxy sequencing (Sambrook et al, 1989). The reporter plasmid, 17M-ERE-globin-CAT, and the expression vectors, GAL-ER(HBD), have been described previously (Webster et al, 1988).

COS-1, HeLa and MCF-7 cells were maintained as described previously (Ali et al, 1993*a*), split into 9-cm plates in Dulbecco's modified Eagle medium (DMEM)–phenol red with 5% double charcoal-stripped FCS and transfected using the calcium phosphate technique (Tora et al, 1989*b*). COS-1 and HeLa cells were transfected with 2 μg of 17M-ERE-globin-CAT along with 0.5 μg of the β-galactosidase reference plasmid, pCH110 (Pharmacia, UK), 0.5 μg of pSG5, HEG0, HE15 or HEΔ5 expression plasmids, together with Bluescribe M13$^+$ DNA (BSM$^+$; Stratagene, UK) as carrier DNA to make a total of 20 μg of DNA. MCF-7 cells were transfected with 2.0 μg of 17M-ERE-globin-CAT and 4 μg of pCH110. Varying amounts of HEΔ5 DNA together with pSG5, to a total of 5 μg, and 9 μg of BSM$^+$ to a total of 20 μg of DNA, were used. Oestrogen, hydroxytamoxifen or ICI 164, 384 were added as appropriate, cells were harvested and CAT assays were performed as described (Ali et al, 1993*a*).

© Cancer Research Campaign 1997 

### Immunoblotting, gel shifts and immunocytochemistry

COS-1 cells were transfected with 5 μg of pSG5 or HEG0 or 10 μg of HEΔ5, together with human placental DNA (Sigma, UK) to a total of 20 μg for immunoblotting, gel shifts and immunocytochemistry, as above. Whole cell extracts were prepared from confluent 9-cm plates of transfected pSG5-, HEG0- and HEΔ5-transfected COS-1 cells for immunoblotting and gel shift assays. Cells were washed with chilled phosphate-buffered saline (PBS), scraped, collected in PBS, centrifuged at 1000 ***g*** for 5 min at 4°C, and cell pellets were resuspended in 100 μl of 20 mM Tris-HCl, pH 7.5, 400 mM potassium chloride, 2 mM dithiothreitol, 1 mM EDTA, 20% glycerol, 0.5 mM phenylmethylsulphonyl fluoride (PMSE) and 0.5 μg $ml^{-1}$ leupeptin, aprotinin, pepstatin, antitrypsin and chymostatin. After three cycles of freeze–thaw (–80°C/0°C), the samples were centrifuged at 15 000 ***g*** for 20 min at 4°C and stored at –80°C until required.

COS-1 extracts (10 μg) were resolved on 10% sodium dodecyl sulphate polyacrylamide gel electrophoresis (SDS-PAGE) and immunoblotting was performed essentially as described (Ali et al, 1993*b*). The ER monoclonal antibody, B10 (Ali et al, 1993*b*), was used at 0.5 μg $ml^{-1}$ and αHEΔ5 was used at 2 μg $ml^{-1}$ for immunoblotting. Alkaline phosphatase-labelled rabbit anti-mouse IgG was used as the second antibody and visualisation was carried out using BCIP and NBT substrates as indicated in the manufacturer's protocol (Promega, UK).

Gel shift assays were performed using 5 μg of the COS-1 extracts as described (Ali et al, 1993*b*). For 'supershifts' 1–2 μg of B10, F3 and αHEΔ5 were added to the gel shift mix.

For immunocytochemistry, COS-1 cells were grown on glass coverslips in 9-cm dishes. Transfections were performed as above. Cells were fixed in 3.7% formaldehyde–PBS for 10 min, washed with PBS for 5 min, placed in methanol for 3 min at –20°C, cold acetone for 1 min at –20°C and washed twice with PBS for 5 min. The coverslips were incubated in 10% goat serum in PBS for 15 min to block non-specific binding. Primary antibody (at 0.5 μg $ml^{-1}$) was added and incubation carried out for 1 h at room temperature followed by two washes in PBS for 5 min. The second antibody (goat anti-mouse IgG; Sigma, UK) was used at 1:25 dilution in PBS for 20 min at room temperature, followed by two washes in PBS. Horseradish peroxidase-labelled mouse anti-goat IgG (Promega, UK) was used in the third incubation at room temperature for 20 min at a dilution of 1:50 in PBS. The coverslips were washed twice in PBS and DAB substrate/reagent (Abbott, UK) was added for 10 min. The coverslips were washed in gently running water for 5 min, counterstained in 1% Harris's haemotoxylin for 5 min and placed twice for 2 min in 95% ethanol, 100% ethanol, then in xylene and mounted on slides coated with a drop of DPX mountant.

Paraffin sections were immunostained as described (Sannino and Shousha, 1994), using the 1D5 (Dako, UK) and αHEΔ5 antibodies diluted to 0.5 μg $ml^{-1}$.

### RESULTS

### PCR analysis of the exon 5 variant in tumour tissue and in normal breast

Oligonucleotides encoding sequences lying within exons 3/4 and 6 of the human ER gene (see Figure 1A) (Ponglikitmongkol et al, 1988) were used to amplify total RNA isolated from 154 breast cancers and 23 non-malignant breast tissues. PCR conditions were optimized such that measurements were made during the linear phase of amplification for both ER and β-actin (data not shown). In cases in which high input led to product saturation, measurements were repeated using diluted sample. Figure 1B illustrates some of the results. A predominant band corresponding to wild-type ER (419 bp) was seen in PCR products from non-malignant breast samples (lanes 5 and 6). Identity was confirmed by digestion of the product with *Hind*III, which produced two fragments of about 160 bp and 270 bp as predicted from the hER cDNA sequence (Green et al, 1986) (data not shown). The PCR products derived from cancer tissue RNA also showed this 419-bp fragment but in about a third of the cases we also observed a smaller 300-bp fragment (Figure 1, lanes 3 and 4), corresponding to the fragment lacking exon 5 sequences described previously (Fuqua et al, 1991). Cloning and sequencing of the two PCR products confirmed the identities of the 419-bp and 300-bp products as wild-type ER and the exon 5-deleted variant respectively (data not shown).

The amount of ER mRNA was determined as a ratio relative to the β-actin levels. None of the tumours were altogether negative for ER transcripts, including those which were ER negative immunohistochemically. Levels of ER–β-actin varied between samples and, in order to determine whether ER transcript levels were proportional to ER protein levels, we compared ER–β-actin ratios with ER status, as determined immunohistochemically. There was a good correlation using the log rank test at 95% CI, with $P$=0.005 (Table 1), indicating that using these PCR conditions the signals obtained reflect real ER mRNA levels in the samples.

Expression of the HEΔ5 variant was found in only 3/23 (13%) non-malignant breast tissues, compared with 50/154 (32%) cancers ($P$=0.05). Altogether 44% of $ER^+/PR^+$ cancers contained HEΔ5, compared with only 15% of $ER^-/PR^-$ cancers. The proportion of $ER^+/PR^-$ and $ER^-/PR^+$ cancers expressing HEΔ5 was not significantly different from the $ER^-/PR^+$ group (32% and 29% respectively)

**Figure 1** The exon 5-deleted oestrogen receptor variant in breast cancer. **A** shows a schematic of the oestrogen receptor mRNA with the positions of exons 1–8 marked. Also shown is the position of the primers used to amplify a region including exon 5. The primer sequences are given in Materials and methods. The predicted amino acid sequence is shown for wild-type ER and the variant (HEΔ5) together with the position of regions A–F. The five amino acids at the C-terminus of HEΔ5 that arise owing to a frameshift are shown in the single letter amino acid code. The asterisk denotes the stop codon leading to premature termination and a truncated polypeptide. **B** shows the PCR products obtained for two breast carcinomas that contain HEΔ5 and two that do not and two normal breast samples. The upper band is the 419-bp product obtained from wild-type mRNA and the lower band (lanes 3 and 4) is the 300-bp variant band. The PCR was performed using 100 ng of RT cDNA and 25 cycles of amplification, as described in Materials and methods

*© Cancer Research Campaign 1997*

**Table 2** Expression of HEΔ5 in tumours in relation to hormone receptor status

| ER/PR | HEΔ5-positive | HEΔ5-negative | Total[a] | Percentage[b] |
|---|---|---|---|---|
| +/+ | 25 | 32 | 57 | 44 |
| +/– | 11 | 23 | 34 | 32 |
| –/+ | 2 | 5 | 7 | 29 |
| –/– | 6 | 35 | 41 | 15 |
| Total | 44 (6) | 95 (8) | 139 (154) | 32 (32) |

ER and PR status were determined immunohistochemically as described in the text and in Materials and methods. [a]PR status was not available for a further 15 tumours; these are included in the figures in brackets. [b]Percentage of tumours positive for HEΔ5 in each class.

(Table 2). The amount of the HEΔ5 product relative to wild-type ER within each sample ranged from 0% to 30% (data not shown). The presence of the HEΔ5 variant was related to both ER ($P$=0.015) and PR ($P$=0.02) status, as determined by immunostaining (Table 1). The presence and absence of HEΔ5 was compared with clinical features in Table 3. There were no apparent correlations between any of the clinical features examined and the presence or absence of HEΔ5, with the exception of menopausal status; 21/51 (41%) of pre-menopausal patients being HEΔ5 positive compared with 29/74 (28%) of post-menopausal patients (but see below).

### Correlation between HEΔ5 levels and clinical parameters

Univariate and multivariate analyses were used to correlate disease-free and overall survival with the presence or absence of HEΔ5 (Tables 4 and 5), compared with other clinical parameters. Using univariate analysis, disease-free and overall survival were not found to be correlated with the age of the patient at the time of diagnosis, or with the menopausal status. There was no correlation with histological features of the cancers, but patients with ER-positive cancers did significantly better than those with ER-negative cancers for both disease-free and overall survival. $PR^+$ patients also showed better overall survival. As expected, clinical and pathological tumour size and nodal status were related to prognosis (Table 4). Interestingly, patients whose tumours contained HEΔ5 appeared to have marginally better disease-free survival than those who did not have any HEΔ5 ($P$=0.05). The presence of HEΔ5 was also associated with increased overall survival, but this failed to reach statistical significance ($P$=0.09). Multivariate survival analysis showed that ER and tumour size were significant independent predictors of disease-free and overall survival; clinical nodal status was found to be significant for disease-free but not overall survival; HEΔ5 was not found to be a statistically significant independent predictor of either disease-free or overall survival. Figure 2 shows survival curves for HEΔ5-positive ($n$=50) and HEΔ5-negative ($n$=104) patients, illustrating a clear trend towards a survival advantage for HEΔ5-positive patients for both disease-free and overall survival.

### Preparation of exon 5-deleted ER-specific monoclonal antibodies

Deletion of sequences encoded within exon 5 of the human oestrogen receptor gene leads to the splicing of exon 4 to exon 6, resulting in a frameshift and the introduction of five new amino acids followed by a stop codon (Figure 1A). Translation of the HEΔ5 mRNA would, therefore, be expected to yield a polypeptide of about 40 kDa. We synthesized a peptide with the sequence GTRENV, corresponding to the final six C-terminal amino acids of HEΔ5 and containing a cysteine at the N terminus for coupling to tuberculin for immunization of mice. The peptide was immunogenic in mice as determined by ELISA and immunoblotting of HEΔ5-transfected COS-1 cell extracts (data not shown). After cell fusion, supernatants from 29 cultures were tested by ELISA, by

**Table 3** Clinical features of patients with respect to HEΔ5 expression

| | HEΔ5-positive | HEΔ5-negative |
|---|---|---|
| Age (years)[a] | | |
| Range | 39–90 | 26–85 |
| Mean | 56.9 | 59.4 |
| Menopausal status | | |
| Premenopausal | 21 (42%) | 30 (29%) |
| Post-menopausal | 29 (58%) | 74 (71%) |
| Total | 50 | 104 |
| Tumour size[b] | | |
| T1–T2 | 32 (82%) | 63 (77%) |
| T3–T4 | 7 | 19 |
| Not known[c] | 11 | 22 |
| Total | 50 | 104 |
| Nodal status | | |
| Positive | 14 (37%) | 37 (42%) |
| Negative | 24 (63%) | 50 (58%) |
| Not known[d] | 12 | 17 |
| Total | 50 | 104 |
| Histology | | |
| Invasive ductal | 46 (92%) | 95 (91%) |
| Invasive lobular | 2 (4%) | 5 (5%) |
| DCIS | 0 | 1 (1%) |
| LCIS | 0 | 0 |
| Others | 2 (4%) | 3 (3%) |
| Total | 50 | 104 |

Fifty cancers were HEΔ5 positive and 104 were HEΔ5 negative. Figures in brackets are percentages calculated using only the known values. [a]Follow-up (mean): 61.2 months. [b]Tumour size was assessed clinically according to TNM staging (*UICC Handbook*). [c]Not accurately assessed clinically. [d]Axilla not dissected.

**Table 4** Univariate survival analysis[a]

| | | Disease-free survival | Overall survival |
|---|---|---|---|
| Age (<50 years vs ≥ 50 years) | | NS[b] | NS |
| ER | Negative | 1.00 | 1.00 |
| | Positive | 0.61[a] (0.39–0.96) $P$=0.03 | 0.46 (0.27–0.78) $P$=0.003 |
| PR | Negative | 1.00 | 1.00 |
| | Positive | 0.73 (0.50–1.06) $P$=0.09 | 0.60 (0.37–0.95) $P$=0.03 |
| V WT ratio | 0 | 1.00 | 1.00 |
| | >0 | 0.62 (0.38–1.01) $P$=0.05 | 0.60 (0.33–1.08) $P$=0.09 |
| Tumour size (T stage) | | Trend ($P$<0.001) | Trend ($P$<0.001) |
| Nodal status (0, 1a, 1b) | | Trend ($P$<0.001) | Trend ($P$<0.001) |
| Histology | | NS | NS |
| Menopausal status (pre/peri–post) | | NS | NS |

[a]Log rank test: hazard ratio (95% CI) ($P$-value) given. [b]NS, not significant at 95% CI. [c]ER-positive patients are only 61% as likely to experience an event as ER-negative patients at any time point during follow-up.

© Cancer Research Campaign 1997

**Table 5** Multivariate survival analysis[a]

| | | Disease-free survival | Overall survival |
|---|---|---|---|
| Age (<50 years vs ≥50 years) | | NS[b] | NS |
| ER | Negative | 1.00 | 1.00 |
| | Positive | 0.55[c] (0.33–0.93) | 0.35 (0.19–0.65) |
| | | *P*=0.03 | *P*=0.001 |
| PR | Negative | 1.00 | 1.00 |
| | Positive | 0.93 (0.57–1.51) | 0.68 (0.36–1.26) |
| | | *P*=0.75 | *P*=0.21 |
| V:WT | 0 | 1.00 | 1.00 |
| | >0 | 0.66 (0.37–1.19) | 0.58 (0.28–1.18) |
| | | *P*=0.16 | *P*=0.12 |
| Tumour size[d] | T1 | 1.00 | 1.00 |
| | T2 | 1.57 | 1.73 |
| | T3 | 2.46 | 2.98 |
| | T4 | 3.85 | 5.14 |
| | | *P*=0.002 | *P*<0.001 |
| Nodal status (positive vs negative) | | 1.52 (1.05–2.2) | NS |
| | | *P*=0.03 | |
| Histology | | NS | NS |
| Menopausal status (pre/peri–post) | | NS | NS |

[a]Performed using Cox's regression allowing for tumour size and nodal status: hazard ratio (95% CI) (*P*-value) given. [b]NS, not significant at 95% CI. [c]ER-positive patients are only 55% as likely to experience an event as ER-negative patients at any time point during follow-up. [d]Tumour size was assessed clinically according to TNM staging (*UICC Handbook*).

immunoblotting and immunocytochemistry of HEΔ5-transfected COS-1 cells. A single hybridoma was selected, based on the intensity and specificity of the reactions obtained in these tests (data not shown; and see below) and identified as IgG2b. It was further amplified, culture supernatants were collected and ascites fluid was produced, and is hereafter referred to as αHEΔ5.

### Detection of HEΔ5 protein by the αHEΔ5 monoclonal antibody in transiently tranfected cells

COS-1 cells were transiently transfected with the plasmid pSG5 or with pSG5 containing either HEG0 or HEΔ5. Cell extracts were prepared and the αHEΔ5 monoclonal antibody was tested for specificity by immunoblotting. The monoclonal antibody, B10, raised against aminoacids 150–165 (region B) of the human oestrogen receptor (Ali et al, 1993*b*) was used for comparison with αHEΔ5. Figure 3A shows that B10 recognized both the wild-type ER (HEG0, lane 2) and a band at about 40 kDa, consistent with the size expected for the HEΔ5 polypeptide (lane 3). αHEΔ5 did not recognize HEG0 but did react with the 40-kDa HEΔ5 polypeptide (compare lanes 2 and 3 with lanes 5 and 6).

The DNA binding of ER to an oestrogen response element (ERE) can be determined in vitro using the gel shift assay by incubating cell extracts containing ER with radioactively labelled ERE-containing oligonucleotides and then resolving the ER–ERE complex using non-denaturing polyacrylamide gel electrophoresis (Ali et al, 1993*b*). This assay also showed that αHEΔ5 recognizes HEΔ5 but does not recognize the wild-type hER (HEG0). Whole cell extracts (WCE) of transfected COS-1 cells expressing pSG5, HEG0 or HEΔ5 were incubated with $^{32}$P-labelled oligonucleotides containing an ERE. WCE of COS-1 cells transfected with the parent vector pSG5 gave no binding to the ERE (Figure 3B, lanes 1, 4, 7 and 10), in contrast to HEG0-transfected COS-1 cells,

**Figure 2** Disease-free and overall survival curves for patients with variant-negative (V:WT = 0) and variant-positive (V:WT > 0) breast carcinomas. **A** shows disease-free survival for the two groups (*n*=97 for variant-negative and *n*=47 for variant-positive patients) Variant (HEΔ5)-positive patients had better disease-free survival (*P*=0.05). **B** shows that these patients also had better overall survival with *P*=0.09 (not significant). The vertical lines show the confidence intervals and the numbers at risk are shown which displayed a prominent retarded band (lane 5). HEΔ5-transfected COS-1 cell extracts also gave a retarded band that migrated slightly faster than the HEG0 complex (compare lanes 5 and 6). Note that the HEΔ5–ERE complex was much weaker than the HEG0–ERE complex, despite similar levels of each protein being present in the extracts as judged by immunoblotting (data not shown; and see Figure 3A, compare lanes 2 and 3).

Addition of the F3 monoclonal antibody, which recognizes amino acids 578–595 of hER (Ali et al, 1993*b*), 'supershifted' the HEG0–ERE complex owing to the formation of a large antibody–ER–ERE complex (compare lanes 2 and 5). The HEΔ5 complex was not supershifted because of the absence of amino acids 578–595 (lane 3). B10 supershifted both HEG0 and HEΔ5 (lanes 8 and 9), as expected. αHEΔ5 did not supershift HEG0 (lane 11) but did give slower migration of HEΔ5 (lane 12), further showing that αHEΔ5 recognizes HEΔ5, but not wild-type, ER.

© Cancer Research Campaign 1997

**Figure 3** Immunoblot gel shift and immunocytochemical analysis of COS-1 cells transfected with ER and HEΔ5 using B10 and αHEΔ5 monoclonal antibodies. (**A**) Lanes 1–3 and 4–6 were immunoprobed with B10 and αHEΔ5 respectively. The positions of the molecular weight standards are indicated in kDa. **B** shows gel shift analysis of pSG5-, HEG0- and HEΔ5-transfected COS-1 cell extracts, in the presence of control antibody (lanes 4–6), F3 (lanes 1–3), B10 (lanes 7–9) or αHEΔ5 (lanes 10–12). (**C**) COS-1 cells transfected with pSG5, HEG0 or HEΔ5 were fixed and incubated with either mouse IgG (control), B10 or αHEΔ5. Counterstaining was performed with haematoxylin

COS-1 cells were grown on coverslips, transfected with pSG5, HEG0 or HEΔ5 using the calcium phosphate method, fixed and immunostained using mouse IgG, B10 and αHEΔ5 antibodies. Figure 3C shows that no staining was observed with pSG5-transfected cells. Both HEG0- and HEΔ5-transfected COS-1 cells showed a proportion of cells with strong nuclear staining. Staining with αHEΔ5 gave nuclear staining only in COS-1 cells transfected with HEΔ5.

### Expression of HEΔ5 protein in breast carcinomas

The above transfection studies showed that αHEΔ5 can specifically recognize the HEΔ5 polypeptide in immunoblotting, gel shift and immunostaining assays. We next examined breast cancer samples for the presence of HEΔ5 protein. Twenty samples were chosen on the basis of high and low levels of HEΔ5 mRNA, as determined by PCR (above). Extracts were prepared from tissue stored in liquid nitrogen by homogenization, followed by freeze–thawing (–80°C/0°C) and centrifugation at 15 000 ***g*** for removal of insoluble material. The lysates were analysed by immunoblotting and gel shift assays. We found no evidence for the presence of the HEΔ5 polypeptide (data not shown).

The reactivity of αHEΔ5 was evaluated by using frozen sections, since immunohistochemistry offers a more sensitive method of detection than the methods used above. Frozen sections from a cancer expressing high levels of HEΔ5 mRNA, as determined by PCR (a ratio of 0.25 variant–wild-type ER; see Figure 1B, lane 3), were immunostained for the presence of HEΔ5 protein. Serial sections were immunostained using the ER monoclonal antibody 1D5 (Dako, UK), which was raised against an epitope in the N-terminal portion of the ER protein, and therefore would be expected to recognize both the wild-type ER and the HEΔ5 polypeptides. 1D5 exhibited strong nuclear staining confined to

*© Cancer Research Campaign 1997*

the carcinoma cells (Figure 4A). αHEΔ5 similarly gave nuclear staining of malignant cells (Figure 4B), although some cytoplasmic staining was also evident. However, the specificity of αHEΔ5 was confirmed by immunostaining with mouse IgG and by competing with the immunizing antigen. Immunostaining with mouse immunoglobulins gave no staining (Figure 4C). Preincubation of αHEΔ5 with the peptide C-GTRENV, used for immunization, led to loss of staining (Figure 4D), whereas 1D5 staining was unchanged (Figure 4E), further indicating that the HEΔ5 staining was specific.

In order to demonstrate further the specificity of our antibody, immunostaining was performed on another cancer positive for ER and for HEΔ5 mRNA expression and one that showed ER positivity but HEΔ5 negativity by PCR. The staining observed for an $ER^+/PR^+$ cancer expressing high levels of HEΔ5 mRNA (variant–WT ratio=0.25; see Figure 1B, lane 4) is shown in Figure 4F–H. Strong nuclear staining of the malignant cells was observed with the ER antibody 1D5 (Figure 4F). αHEΔ5 also showed nuclear staining of some malignant cells, as well as cytoplasmic positivity (Figure 4G). Case 2 gave positive staining for ER (using 1D5; Figure 4I) but no nuclear staining was seen with αHEΔ5 (Figure 4J). PCR results showed that this sample was negative for HEΔ5 mRNA (Figure 1B, lane 1). No staining was observed in any of the cases with mouse IgG (Figure 4H and K).

*© Cancer Research Campaign 1997*

**Figure 4** Detection of HEΔ5 using the αHEΔ5 antibody and comparison with an ER antibody on HEΔ5-positive and -negative breast cancer sections. Sections of a strongly ER- and HEΔ5-positive breast cancer sample were immunostained with 1D5 (**A**), αHEΔ5 (**B**) or mouse IgG (**C**). (**D–E**) shows the staining obtained after incubation of αHEΔ5 (**D**) or 1D5 (**E**) with a 100-fold excess of the immunizing peptide. Another strongly HEΔ5-positive (**F–H**) and a HEΔ5-negative (**I–K**) breast cancer sample were immunostained with the ER antibody 1D5 (**F** and **I**), αHEΔ5 (**G** and **J**) or mouse IgG (**H** and **K**). Counterstaining was performed with haematoxylin. Original magnification × 200

### Transcriptional activation by HEΔ5 in mammalian cells

We wished to examine HEΔ5 activity in mammalian cell systems and used a chloramphenicol acetyl transferase (CAT) gene reporter system in COS-1 and HeLa cells and in the human breast cancer-derived cell line MCF-7, which overexpresses ER. HEΔ5 is truncated at amino acid 365 in ER and would be expected to act through AF-1 in a constitutive manner. We compared the *trans*-activational ability of HEΔ5 with ER (HEG0) and with the in vitro generated mutant HE15 [containing amino acids 1–281 of ER (Kumar et al, 1987], which activates transcription through AF-1 (Tora et al, 1989*a*). The constructs used and the extent of the deletions are shown in Figure 5A.

Transfection of HEG0 into COS-1 cells resulted in a tenfold increase in transcription in the presence of oestradiol (E2) over its absence and a 20-fold increase relative to background (Figure 5B); the *trans*-activation produced by HEG0 in the absence of E2 may be caused by residual oestrogens in the charcoal-stripped medium used (Tora et al., 1989*b*). HE15 activated the CAT gene about 15% as well as HEG0 and showed the same activity in the presence as in the absence of E2 (Figure 5B; and data not shown). HEΔ5 *trans*-activated to levels similar to those obtained with HE15 (Figure 5B).

Several studies have shown that AF-1 and AF-2 of ER are separable and synergise to give maximal *trans*-activation by ER (Lees et al, 1989; Tora et al, 1989*a*). The synergistic activity of AF-1 and AF-2 can be examined using constructs in which AF-2 (region E)

© Cancer Research Campaign 1997

**Figure 5** Comparison of the transcriptional activities of hER, HE15 and HEΔ5 in various cell lines using the 17M-ERE-globin-CAT reporter gene. **A** shows a schematic of the human oestrogen receptor gene, together with its exonic structure. Also shown are HEGO, HEΔ5 and HE15 and the regions present in HEΔ5 and HE15 are indicated. **B** and **C** show transcriptional stimulation of the reporter gene in COS-1 and HeLa cells, respectively, by the wild-type human ER (HEGO), the truncated ER mutant HE15, the exon 5 variant HEΔ5 or GAL-ER (HBD) in the absence or presence of $10^{-8}$M oestradiol (E), $10^{-7}$M 4-hydroxytamoxifen (O) and $10^{-7}$M ICI 164, 384 (I), as indicated. (**D**) MCF-7 cells were transfected with the ERE-globin-CAT reporter gene with or without increasing amounts of HEΔ5, in the presence or absence of E, O or I. Average values of at least three experiments are shown, the level of *trans*-activation by HEGO in the presence of E being taken as 100% in each experiment is fused to the DNA-binding domain of the yeast *trans*-activator GAL4 (GAL-ER (HBD)), and using HE15 in which AF-2 sequences have been deleted. When co-transfected together with a CAT reporter gene containing a GAL4 DNA-binding element (17M) and an ERE, AF-1 and AF-2 act synergistically (Webster et al, 1988; Tora et al, 1989*a*). In order to examine the mechanisms of *trans*-activation by HEΔ5 further and to determine whether it *trans*-activates ERE-dependent gene expression in a similar way to HE15, i.e. through AF-1, we analysed its ability to synergise with AF-2 (Figure 5B). GAL-ER activated the 17M/ERE-G-CAT gene in the presence of E2. Both HE15 and HEΔ5 synergised with GAL-ER to similar extents.

In HeLa cells, AF-1 is unable to activate transcription but can synergise with AF-2 (Kumar et al, 1987; Lees et al, 1989; Tora et al, 1989*a*; Berry et al, 1990). Both HE15 and HEΔ5 gave very little *trans*-activation on their own but synergised with GAL-ER in the presence of E2 (Figure 5C). These results indicate that HEΔ5 acts in a similar manner to HE15, i.e. through AF-1, although as before 4.0 µg of HEΔ5 needed to be transfected compared with 0.5 µg of HE15 for this level of synergism.

Tamoxifen (and its metabolite, 4-hydroxy-tamoxifen) is a partial antagonist of oestrogen, in that it enables *trans*-activation through AF-1, while inhibiting the activity of AF-2 (Berry et al, 1990). ER *trans*-activated about 20% as well in the presence of hydroxy-tamoxifen (O) as in the presence of E2 in COS-1 cells, whereas little activity was seen in HeLa cells. HE15 and HEΔ5 also *trans*-activated in COS-1 cells but not in HeLa cells. These results are in broad agreement with those reported previously (Berry et al, 1990). Note also that, in the presence of hydroxy-tamoxifen, little synergistic activity was observed when GAL-ER and HE15 or HEΔ5 were co-transfected. As expected, in the presence of the 'pure' antagonist, ICI 164,384, very little ER activity was observed.

In order to examine the effect of HEΔ5 on *trans*-activation in the presence of wild-type ER, MCF-7 cells were transfected with HEΔ5 and the CAT reporter gene. We observed a fivefold induction by the endogenous ER, on addition of E2, in cells transfected with the CAT reporter alone. Co-transfection with increasing amounts of HEΔ5 inhibited this *trans*-activation by up to 60% indicating that HEΔ5 was acting as a 'dominant negative' mutant, presumably by virtue of its lower *trans*-activational ability compared with the wild-type ER. Note, however, that the inhibition never reached levels obtained with hydroxy-tamoxifen (Figure 5D). An inhibition by HEΔ5 of the low level *trans*-activation in the absence of ligand and in the presence of hydroxy-tamoxifen and ICI 164,384 was also observed and might suggest that, as in HeLa cells, HEΔ5 cannot *trans*-activate on its own from this promoter.

### DISCUSSION

The exon 5 deletion variant was first shown to be present at the mRNA level in ER$^-$/PR$^+$ breast cancers by Fuqua et al (1991), who showed that it could *trans*-activate constitutively in a yeast expression system. We have analysed 154 breast cancers, chosen without regard to their clinical status, for the presence of HEΔ5 by PCR. We have shown that the amount of wild-type ER product obtained by this method was strongly correlated with the presence of ER as determined immunohistochemically (at 95% CI, $P$=0.005). HEΔ5

© Cancer Research Campaign 1997

was present in about 30% of the cancers but in only 10% of normal breast tissue ($P$=0.05). In breast cancer, the presence of HEΔ5 was related to the presence of ER (at 95% CI, $P$=0.015) and PR ($P$=0.02), in agreement with other data (Daffada et al, 1995). Prognostic tests using univariate analysis showed that the presence of HEΔ5 is associated with longer disease-free survival, although the results only reach significance to 95% CI with $P$=0.05. Multivariate survival analyses showed that the presence of HEΔ5 is not an independent prognostic factor. These results were unexpected in view of suggestions that HEΔ5 could lead to resistance to endocrine therapy, but concur with the results of Daffada et al (1995), who showed similar levels of HEΔ5 in tamoxifen-resistant cancers and primary controls.

A number of studies have reported the presence of ER variants at the mRNA level but none have provided evidence that these mRNAs are translated. Several studies have found evidence for the presence of ER-like proteins unable to bind DNA or ligand but these could be proteolytic products of wild-type ER protein. We therefore raised a monoclonal antibody that would specifically recognize the HEΔ5 polypeptide and present the first evidence for the presence of HEΔ5 protein in breast tissues. In an immunohistochemical study, we have shown that tumours that possess the HEΔ5 mRNA have immunostainable HEΔ5 protein. Although we have demonstrated the specificity of our antibody by artificially overexpressing HEΔ5 in COS-1 cells, we have not yet been able to demonstrate its presence in breast cancer using immunoblotting or gel shift assays, presumably because of low amounts of endogenous HEΔ5 protein and/or its reduced stability. Further studies are underway to compare the stability of wild-type and exon 5-deleted ER proteins using pulse-chase labelling experiments.

We have, furthermore, analysed the *trans*-activational ability of HEΔ5 by transient transfection of the ER-negative COS-1 and HeLa cell lines and in the ER-positive MCF-7 cells. We find that HEΔ5 can constitutively *trans*-activate ERE-dependent gene expression in mammalian cells in addition to the previously described activity in yeast (Fuqua et al, 1991). HE15, which contains the DBD and AF-1 of the human oestrogen receptor but lacks the HBD/AF-2, has been well characterized (Kumar et al, 1986, 1987). This construct has been extensively used to show that AF-1 activity is cell- and promoter-specific (Tora et al, 1989*a*; Berry et al, 1990; Metzger et al, 1995). Furthermore, the activity of the wild-type ER in the presence of tamoxifen has been correlated to the activity of HE15 by comparing the cell and promoter specificity of *trans*-activation by hER in the presence of tamoxifen, indicating that tamoxifen inhibits AF-2 but allows activation of AF-1 (Berry et al, 1990). The experiments outlined in Figure 5 indicate that HEΔ5 behaves mechanistically like HE15 (amino acids 1–281 of ER), which only contains AF-1 of ER since: (1) HEΔ5 can activate a reporter gene in COS-1 cells to similar levels as HE15; but (2) like HE15, HEΔ5 does not *trans*-activate in HeLa cells (see Kumar et al, 1987; Tora et al, 1989*a*; Berry et al, 1990); (3) HEΔ5 can synergise with AF-2 (in GAL-ER) in COS-1; and (4) despite being inactive on its own in HeLa cells, HE15 is capable of synergising with AF-2 (GAL-ER (HBD)), as is HEΔ5. Given our findings that HEΔ5 shows similar cell specificity and synergistic properties to HE15, our results indicate that the constitutive *trans*-activation by HEΔ5 results entirely from AF-1 sequences and, since the agonistic activity of tamoxifen is caused by its activation of AF-1, while inhibiting AF-2, it is unlikely that resistance to tamoxifen should arise as a result of the induction of, or increase in levels of, an ER variant with the same *trans*-activational properties as the full-length ER bound by tamoxifen.

Transfection of MCF-7 cells showed that HEΔ5 inhibits *trans*-activation by the endogenous receptor, presumably by competitive binding on EREs in responsive genes owing to its lower *trans*-activational ability relative to the wild-type receptor. In this respect, HEΔ5, despite its ability to *trans*-activate constitutively, actually inhibits the action of ER, thereby acting in a manner similar to some anti-oestrogens, and should not be expected to be responsible for resistance to tamoxifen therapy, as has been suggested (see Introduction). The analysis of pre- and post-therapy biopsy samples for changes in the level of the HEΔ5 variant may shed more light on this issue. Fuqua (1994) reported that transfection of HEΔ5 into MCF-7 cells makes them resistant to tamoxifen. These results are in apparent contradiction to our results showing that HEΔ5 inhibits *trans*-activation by ER, although we have performed these studies using chimeric reporter genes. We found the greatest levels of HEΔ5 mRNA in $PR^+$ cancers and the lowest in $PR^-$ cancers, regardless of ER status (data not shown). Similarly a study describing the analysis of 27 breast cancers (Zhang et al, 1993) for the presence of HEΔ5 reported highest levels of HEΔ5 in $PR^+$ cancers. In this series only 1/13 $ER^+/PR^+$ and 0/3 $ER^-PR^+$ cancers failed to express HEΔ5, whereas 3/7 $ER^+/PR^-$ and 4/4 $ER^-/PR^-$ cancers did not express HEΔ5. Another study (Daffada et al, 1995) recently showed correlation of pS2 and PR status and the presence of HEΔ5. Taken together, these results suggest that these ER-responsive genes might be regulated, at least in part, by HEΔ5. The correlation between the presence of HEΔ5 and the presence of PR and pS2 is in agreement with our results indicating better prognosis for HEΔ5-positive cancers, as these indicators have themselves been shown to be correlated with a better prognosis (Ravdin et al, 1992; Foekens et al, 1993).

In conclusion, we find that the HEΔ5 variant is present in a proportion of breast cancers at the mRNA level and that its presence does not correlate with a poor prognosis. Its *trans*-activational properties suggest that it inhibits *trans*-activation by wild-type ER, most likely by direct competition. Our results do, however, support the notion suggested by others that HEΔ5 could play a role in regulating the expression of certain genes. The detection of the HEΔ5 polypeptide in breast cancers using a specific antibody is further indication that HEΔ5 plays a role in breast cancer.

### ADDENDUM

Rea and Parker (1996) recently reported the creation of MCF-7 cell lines stably expressing HEΔ5 and showed that the variant stimulated transcription of a reporter gene in chicken embryonal fibroblasts in the absence of hormone but was only weakly active in MCF-7 cells. They further showed that the growth-stimulatory effects of oestrogen and the growth-inhibitory effects of tamoxifen were not influenced by the presence of HEΔ5. These findings are in general agreement with our results indicating that HEΔ5 is not responsible for resistance to endocrine therapy in breast cancer.

### ACKNOWLEDGEMENTS

We thank Professor Pierre Chambon for his generosity with expression plasmids and reporter constructs, Roger A'Hern for performing the statistical analyses and Eke Engstrom for mass spectrometric analysis of the peptide. ICI 164,384 was kindly provided by Alan Wakeling (Zeneca). This research was funded by grants from the Cancer Research Campaign and the Medical Research Council.

© Cancer Research Campaign 1997

### REFERENCES

Aldwin L and Nitecki DE (1987) A water soluble, monitorable peptide and protein crosslinking agent. *Anal Biochem* **164**: 494–501

Ali S, Metzger D, Bornert J-M and Chambon P (1993*a*) Phosphorylation of the human oestrogen receptor; identification of a phosphorylation site required for trans-activation. *EMBO J* **12**: 1153–1160

Ali S, Lutz Y, Bellocq J-P, Chenard-Neu M-P, Rouyer N and Metzger D (1993*b*) Production and characterisation of monoclonal antibodies recognising defined regions of the human oestrogen receptor. *Hybridoma* **12**: 391–405

Atherton E and Sheppard RC (1985) Solid phase peptide synthesis using N. fluorenyl methocarboxycarbonyl amino-acid penta fluorophenyl esters. *J Chem Soc Chem Commun* **15**: 165–166

Bansal GS, Yiangou C, Coope RC, Gomm J, Johnston C, Coombes RC and Luqmani YA (1995) Expression of fibroblast growth factor 1 in human breast. *Br J Cancer* **72**: 1420–1426

Barrett-Lee PJ, Travis M, McClelland RA, Luqmani Y and Coombes RCC (1987) Characterisation of estrogen receptor messenger RNA in human breast cancer. *Cancer Res* **47**: 6653–6659

Berry M, Metzger D and Chambon P (1990) Role of the two activating domains of the estrogen receptor in the cell-type and promoter-context dependent agonistic activity of the anti-oestrogen 4-hydroxytamoxifen. *EMBO J* **9**: 2811–2818

Burgess HE and Shousha S (1993) An immunohistochemical study of the long-term effects of androgen administration on female-to-male transsexual breast: a comparison with normal female breast and male breast showing gynaecomastia. *J Pathol* **170**: 37–43

Daffada AI, Johnston SRD, Smith IE, Detre S, King N and Dowsett M (1995) Exon 5 deletion variant estrogen receptor messenger RNA in relation to tamoxifen resistance and progesterone receptor/pS2 status in human breast cancer. *Cancer Res* **55**: 288–293

Dotzlaw H, Alkhalaf M and Murphy LC (1992) Characterisation of estrogen receptor variant mRNAs from human breast cancer. *Mol Endocrinol* **6**: 773–785

Foekens JA, Van-Putten WL, Portengen H, De-Koning HY, Thirion B, Alexieva-Figusch J and Klijn JG (1993) Prognostic value of PS2 and cathepsin D in 710 human primary breast tumours: multivariate analysis. *J Clin Oncol* **11**: 899–908

Foster BD, Cavener DR and Parl FF (1991) Binding analysis of the estrogen receptor to its specific DNA target site in human breast cancer. *Cancer Res* **51**: 3405–3410

Fuqua Saw (1994) Estrogen receptor mutagenesis and hormone resistance. *Cancer* **74**: 1026–1029

Fuqua SAW, Fitzgerald SD, Chamness GC, Tandon AK, McDonnell DP, Nawaz Z, O'Malley BW and McGuire WL (1991) Variant human breast tumor estrogen receptor with constitutive transcriptional activity. *Cancer Res* **51**: 105–109

Fuqua SAW, Fitzgerald SD, Allred C, Elledge RM, Nawaz Z, McDonnell DP, O'Malley BW and McGuire WL (1992) Inhibition of estrogen receptor action by a naturally occuring variant in human breast tumors. *Cancer Res* **52**: 483–486

Galfre G and Milstein C (1987) Preparation of monoclonal antibodies: strategies and procedures. *Methods Enzymol* **73**: 3–36

Green S and Chambon P (1987) Oestradiol induction of a glucocorticoid-responsive gene by a chimaeric receptor. *Nature* **325**: 75–78

Green S, Walter P, Kumar V, Krust A, Bornert J-M, Argos P and Chambon P (1986) Human estrogen receptor cDNA: sequence, expression and homology to v-erbA. *Nature* **320**: 134–139

Harlow E and Lane D (1988) *Antibodies: A Laboratory Manual*. pp. 553–613. Cold Spring Harbor Laboratory Press: New York

Horwitz KB (1994) How do breast cancers become hormone resistant? *J Steroid Biochem Mol Biol* **49**: 295–302

King DS, Fields CG and Fields GB (1990) A cleavage method which minimizes side reactions following Fmoc solid phase peptide synthesis. *Int J Peptide Protein Res* **36**: 255–266

Krust A, Green S, Argos P, Kumar V, Walter P, Bornert J-M and Chambon P (1986) The chicken oestrogen receptor sequence: homology with v-erbA and the human oestrogen and glucocorticoid receptors. *EMBO J* **5**: 891–897

Kumar V, Green S, Staub A and Chambon P (1986) Localisation of the oestradiol-binding and putative DNA-binding domains of the human oestrogen receptor. *EMBO J* **5**: 2231–2236

Kumar V, Green S, Stack G, Berry M, Jin JR and Chambon P (1987) Functional domains of the human oestrogen receptor. *Cell* **51**: 941–951

Lachmann PJ, Strangeways L, Vgakurum A and Evans GI (1986) In *Synthetic Peptides as Antigens*. Ciba Symposium V119 pp. 25–40. Wiley: Chichester

Lees JA, Fawell SE and Parker MG (1989) Identification of two trans-activation domains in the mouse oestrogen receptor. *Nucleic Acids Res* **17**: 5477–5488

Lehrer S, Sanchez M, Song HK, Dalton J, Levine E, Savoretti P, Thung SN and Schachter B (1990) Oestrogen receptor B-region polymorphism and spontaneous abortion in women with breast cancer. *Lancet* **335**: 622–624

Lehrer S, Rabin J, Kalir T and Schachter BS (1992) Estrogen receptor variant and hypertension in women. *Hypertension* **21**: 439–441

McCullough KC and Spier RE (1990) *Monoclonal Antibodies in Biotechnology: Theoretical and Practical Aspects*. pp. 258–261. Cambridge University Press: Cambridge, UK

McGuire WL (1978) Hormone receptors: their role in predicting prognosis and response to endocrine therapy. *Semin Oncol* **5**: 428–433

McGuire WL, Chamness GC and Fuqua SAW (1991) Estrogen receptor variants in clinical breast cancer. *Mol Endocrinol* **5**: 1571–1577

Metzger D, Ali S, Bornert JM and Chambon P (1995) Characterization of the amino-terminal transcriptional activation function of the human estrogen receptor in animal and yeast cells. *J Biol Chem* **270**: 9535–9542

Montano MM, Muller V, Trobaugh A and Katzenellenbogen BS (1995) The carboxy-terminal F domain of the human estrogen receptor: role in the transcriptional activity of the receptor and the effectiveness of antiestrogens as estrogen antagonists. *Mol Endocrinol* **9**: 814–825

Morrison CA, Fishleigh RV, Ward DJ and Robson B (1987) Computer aided design and physiological testing of lutenising hormone-releasing hormone analogue for 'adjuvant free' immunocastration. *FEBS Lett* **214**: 65–70

Murphy LC (1990) Estrogen receptor variants in human breast cancer. *Mol Cell Endocrinol* **74**: C83–C86

Ponglikitmongkol M, Green S and Chambon P (1988) Genomic organisation of the human oestrogen receptor gene. *EMBO J* **7**: 3385–3388

Ravdin PM, Green S, Dorr TM, McGuire WL, Fabian C, Pugh RP, Carter RD, Rivkin SE, Borst JR, Belt RJ, Metch B and Osborne CK (1992) Prognostic significance of progesterone receptor levels in estrogen receptor-positive patients with metastatic breast cancer treated with tamoxifen: results of a prospective Southwest Oncology Group study. *J Clin Oncol* **10**: 1284–1291

Rea D and Parker MG (1996) Effects of an exon 5 variant of the estrogen receptor in MCF-7 breast cancer cells. *Cancer Res* **56**: 1556–1563

Sambrook J, Fritsch EF and Maniatis T (1989) *Molecular Cloning: a Laboratory Manual*. pp. 7.19–7.22. Cold Spring Harbor Laboratory Press: New York

Sannino P and Shousha S (1994) Demonstration of oestrogen receptors in paraffin wax sections of breast carcinoma using the monoclonal antibody 1D5 and microwave oven processing. *J Clin Pathol* **47**: 90–92

Scott GK, Kushner P, Vigne J-L and Benz CC (1991) Truncated forms of DNA binding estrogen receptors in human breast cancer. *J Clin Invest* **88**: 700–706

Sluyser M (1994) Role of estrogen receptor variants in the development of hormone resistance in breast cancer. *Clin Biochem* **25**: 407–414

Smith EP, Boyd J, Frank GR, Takahashi H, Cohen RM, Specker B, Williams TC, Lubahn DB and Korach KS (1994) Estrogen resistance caused by a mutation in the estrogen-receptor gene in a man. *N Engl J Med* **331**: 1056–1061

Tora L, White J, Brou C, Tasset D, Webster N, Scheer E and Chambon P (1989*a*) The human estrogen receptor has two independent nonacidic transcriptional activation functions. *Cell* **59**: 477–487

Tora L, Mullick A, Metzger D, Ponglikitmongkol M, Park I and Chambon P (1989*b*) The cloned human oestrogen receptor contains a mutation which alters its hormone binding properties. *EMBO J* **8**: 1981–1986

Wang Y and Miksicek RJ (1991) Identification of a dominant negative form of the human estrogen receptor. *Mol Endocrinol* **5**: 1707–1715

Webster NJG, Green S, Jin JR and Chambon P (1988) The hormone binding domains of the estrogen and glucocorticoid receptors contain an inducible transcription activation function *Cell* **54**: 199–207

Zhang Q-X, Borg A and Fuqua SAW (1993) An exon 5 deletion variant of the estrogen receptor frequently co-expressed with wild-type estrogen receptor in human breast cancer. *Cancer Res* **53**: 5882–5884

© Cancer Research Campaign 1997